# THE USE OF THE INTERNET BY THE PATIENT AFTER BARIATRIC SURGERY: CONTRIBUTIONS AND OBSTACLES FOR THE FOLLOW-UP OF MULTIDISCIPLINARY MONITORING

**DOI:** 10.1590/S0102-6720201500S100014

**Published:** 2015-12

**Authors:** Michele Pereira MARTINS, Marcela ABREU-RODRIGUES, Juciléia Rezende SOUZA

**Affiliations:** Brasília Institute for Behavior Analysis, Brasília, DF, Brazil.

**Keywords:** Internet, Bariatric surgery, Multidisciplinary monitoring

## Abstract

**ABSTRACT:**

**Background:**

: Bariatric surgery is presented as the last treatment option for obesity. It requires from all candidates a multidisciplinary evaluation and monitoring throughout treatment. The non-adherence to follow-up with health care teams is related to weight regain. It's possible that the use of internet influences the doctor-patient relationship and patients replace medical care or information provided by health professionals for information from the internet.

**Aim:**

: Identify and analyze the pattern of internet use by patients after bariatric surgery and check the influence of such use in attending medical appointments with the multidisciplinary team.

**Method:**

: Electronic questionnaire available on the Internet was used to verify patient´s patterns of Internet use and its influence on in attending multidisciplinary care after surgery.

**Results:**

: Of the 103 participants, 95% were female, 64% married, 59% with children and 54% with higher education. The mean age was 35.69 years and the mean duration of performing surgery, 11.74 months. The surgical technique that prevailed was Roux-en-Y gastric by-pass (90.3%), the local monitoring concentrated in the private care (93.2%). In the preoperative, most participants consulted more than three times with the surgeon (n=81), nutritionists (n=70), psychologist (n=70). After the surgery, p most patients maintained monitoring with the surgeon and nutritionist. Concerning the internet use, 51.5% accessed the internet in search of information about health and bariatric surgery every day. Facebook and search tools were the most used sites.

**Conclusion:**

- Data showed the influence of the information contained on the Internet and the adherence to multidisciplinary monitoring. This fact requires the team to consider the use of the Internet as a variable that may interfere and must be handled during follow-up. It is suggested that an active participation of professionals on their websites and social networks and the diversification of services and interventions to stimulate follow-up after surgery.

## Abstract (pt)

**Racional:**

: O candidato à cirurgia bariátrica precisa passar por avaliação e acompanhamento multiprofissional durante todo o processo de tratamento. Perder o seguimento com as equipes de saúde tem relação com reganho de peso e deficiências nutricionais. O uso da internet é considerado como um dos fatores que pode afetar a relação médico-paciente.

**Objetivo:**

: Identificar e analisar o padrão de uso da internet pelos pacientes no pós-operatório e verificar se influencia no seguimento multiprofissional.

**Metódo:**

: Utilizou-se um questionário eletrônico, disponibilizado on-line, para verificar o padrão de uso da Internet e seu impacto no acompanhamento multiprofissional após o procedimento cirúrgico.

**Resultados:**

: Participaram 103 pacientes, em sua maioria mulheres (95%), casados (64%) e com ensino superior (54%). A idade média foi 35,69 anos e o tempo médio desde a operação de 11,74 meses. Prevaleceram o uso da técnica by-pass gástrico em Y-de-Roux (90,3%), e o acompanhamento foi em serviço privado (93,2%). No pré-cirúrgico a maioria teve maior número de consultas com os profissionais de saúde do que no pós-cirúrgico, havendo relatos que indicam substituição do acompanhamento por informações contida na internet e participação em grupos de discussão on-line, principalmente pela perda de peso inicial gerar sensação de segurança e percepção de não haver mais necessidade de auxílio. O padrão de uso da internet que sobressaiu foi o acesso diário (51,5%) para buscar informações sobre saúde e cirurgia bariátrica. O Facebook e as ferramentas de pesquisa foram os locais mais utilizados.

**Conclusão:**

: Os dados evidenciaram que as informações contidas na Internet influenciam o seguimento multiprofissional, fato este que exige a equipe considerar o uso da rede como variável que interfere e deve ser manejada. Sugere-se a participação ativa dos profissionais em seus sites e redes sociais e a diversificação de intervenções e serviços para estimular o acompanhamento após a operação.

## INTRODUCTION

The operated obese people may have physical and psychological complications. The physical may range from those related to the surgery itself (vomiting, esophageal pinching, dumping syndrome, reactive hypoglycemia, hair loss) to later problems (protein and energy malnutrition, anemia, various hypovitaminosis). Psychological may involve triggering adjustment disorders, severe and chronic psychiatric disorders, eating disorders, alcoholism, impulsive behavior and depression to minimize these difficulties and maximize results, it is necessary that bariatric patients maintain, continuously, in multidisciplinary approach with specialized and well trained staff^1^^,^ 9 ^,^ 14 ^,^ 16.

Preoperatively, the professionals assistance basically has two major focuses. The first includes assessment, diagnosis and treatment of associated diseases, to reduce surgical risk and possible complications. The second concerns the preparation and orientation of the patient on the necessary care in the pre- and postoperative period, as well as to cope with the changes in habits and lifestyle required for treatment^4^^,^ 15.

Psychology is one of the areas responsible for monitoring patients indicated for bariatric surgery, accounting for their evaluation and preparation from the beginning of treatment. From the psychological evaluation is possible to identify and prepare contingency plans to manage psychiatric conditions and or emotional characteristics that could compromise the surgical treatment and rehabilitation^1^^,^ 10. The main goal of psychological preparation is to mobilize the patient to be active and co-responsible for the success of his/her treatment and develop strategies to help to deal with the treatment and make changes in daily habits^1^^,^ 6 ^,^ 10 ^,^ 20.

Postoperatively, is recommended that the multidisciplinary approach should be systematic and frequent in the first month and evolve gradually, respecting the demands of each patient, for monthly, quarterly and semi-annual consultations in the first two years. After this period, it is stated that, throughout their lives, there is the need of annual visits to all specialties^4^^,^ 15. Annual check-up is indicated as one of the most efficient ways for the operated patient to control the weight. Recent studies showed that loss to follow-up with the health teams, reality in the postoperative period, is related to weight regained^2^^,^ 16.

The growth of almost 90% in the number of bariatric operations in Brazil over the past five years - reaching 72,000 in 2012^8^ - also justifies investment in research not only on the regained weight, but on the surgical treatment as a whole. Almino^11^justifies this increase by the patient's use of internet. However, as emphasized by the literature, this access to information also interferes in doctor-patient relationship. The increased use of the internet as a source of health information has been justified by the fact that health represent currently one of the leading man's concerns. Also contribute the increase in the educational level of the population and the number of computers with internet access; the fact of being less costly financially seeking information it compared the cost of using health care services; the convenience and comfort of accessing a multitude of sources, from various places, in various periods and at unprecedented speed^11^.

When there is proper use of information obtained on the Internet, patients, families and professionals can be benefited. Both can facilitate the adoption of a more active attitude in the treatment of the patient, as contribute to the process of communication with health professionals, promoting the sharing of decisions and understanding of health. Still, the patient can get social support by creating and/or participation in virtual communities and support groups^3^^,^ 5 ^,^ 11 ^,^ 12 ^,^ 18 ^,^ 19.

On the other hand, the inappropriate use of the internet can present some dangers: a) incomplete access, contradictions, inaccuracy, fraudulent and even compromised instructions, due in part to the conflict of interest between scientific evidence and marketing strategies of large companies; b) trigger somatic symptoms or psychological interference by misunderstanding the informations or by contact with false information; c) facilitate auto-medication; d) believes that it is possible to replace health care or information provided by health professionals, for information from the internet; e) prioritize the search for information online, instead of doctor visits^35,^ 7 ^,^ 11 ^,^ 12 ^,^ 17 ^,^ 18 ^,^ 19.

The objective of this study was to identify and analyze internet usage pattern of bariatric patients after the surgery, and investigate its influence on the loss in multi-monitoring, specifically in attending medical appointments with the team members.

## METHOD

This study was approved by the Research Ethics Committee - FEPECS / SES-DF (Opinion: 328,626). It is descriptive, mixing qualitative and quantitative methodology, which involved 103 patients undergoing bariatric surgery in public and private institutions around the country. Informations included were the operation was carried out, timing from at least three months and at most two years, both genders, aged 18-65 years and literate.

To collect the information was prepared an electronic questionnaire (EQ), using the Survey Monkey tool, a Web-based application that lets you create and publish online questionnaires for use in research. Such EQ was composed of two home pages; the first contained the consent form and the second clarified instructions for proper filling out the questionnaire. Only after the signing of the term, the survey questions were accessed. The EQ was composed of open and multiple choice questions, which were divided into three focuses: 1) personal data, including demographic and socioeconomic characteristics; 2) data concerning the operation to gather information about the procedure and the multi-monitoring before and after surgery; 3) data on the pattern of internet use and identification of variables related to loss of follow-up by the patient.

The study was conducted in three stages: dissemination of research; publication of EQ; and data analysis. The disclosure of the survey took place between July 8 and August 10, 2013. It was used as outreach strategies direct contact, in which the authors sent the EQ to potential participants, and indirect, in which they sent to professionals who could spread questionnaire for patients, but also to their own patients for divulging among their peers.

## RESULTS

 During data collection, 219 questionnaires were initiated. Of those, 135 were completed, but only 103 met the established inclusion criteria. Among the reasons to invalidate the questionnaires were operation realized less than three or more than 24 months and incorrect reporting of personal data. Only one participant selected the "exit survey", saying that was still waiting for the operation. Probably accessed the EQ for information. Thus, considering the 219 started and 103 valid questionnaires, was achieved response rate around 47%. As there was no control over the amount of disseminated questionnaires, there is no way to know exactly how many patients had access to research.

The average age of the sample was 35.69 years (SD=8.54) and the average time of completion of the operation of 11.74 (SD=6.21) months. The sample included participants from various locations, with half of them being from Federal District (50.5%), probably because the research dissemination of started and remained more concentrated in this region. The technique was most accomplished Roux-en-Y gastric bypass (90.3%, n=93) and most participants was operated on private health care. As can be seen in Table 1, most of the sample were women, married or in a stable union with children. With regard to other characteristics, predominant participants were well-educated, family income higher than 4.1 minimum wages and performing some remunerated labor activity.

**TABLE 1:** - Socio-demographic characterization of the participants (n=103)

**Socio-demographic features**	**n**	**%**	
Gender	Female	98	95,0
	Male	5	5,0
Marital status	Married or stable union	66	64,1
	Single	27	26,2
	Separated/Divorced	8	7,7
	Widow	2	1,9
Sons	No	42	40,8
	Yes	61	59,2
Education	High school	21	20,4
	Superior incomplete	20	19,4
	Superior complete	24	23,3
	Post-graduation	38	37,0
Labor situation	Public server	32	31,1
	Steady job with or without CLT	38	36,9
	Autonomous and/or temporary	18	17,5
	Unemployed	10	9,7
	Never worked	2	1,9
Family income	Less than 1 minimum wage	30	29,1
	From 4,1 to 8 minimum wage	34	33,0
	Mores than 8,1 minimum wage	29	37,9

**FIGURE 1:**
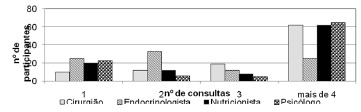
- Pre-surgical frequency of consultations with the multidisciplinary team

Regarding the pre-surgical multi-professional follow-up (Figure 1), most patients consulted more than four times with the surgeon (60.2%), nutritionist (60.2%) and psychologist (63.1%). With the endocrinologist only 24.3% had more than four visits. Compared to patients who underwent only a consultation with professionals before the operation, it was identified the following frequency: surgeon (9.7%), endocrinologists (24.3%), nutritionist (19.4%) and psychologist (22.3%). Other professionals consulted before the operation were: pulmonologist, cardiologist, psychiatrist, physical therapist, speech pathologist, anesthetist, orthopedist, gynecologist and dermatologist.

**FIGURE 2:**
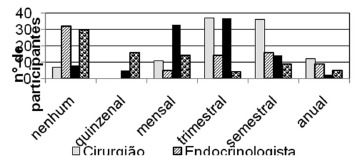
- Post-surgical frequency of consultations with the multidisciplinary team

After surgery (Figure 2) 35.9% held quarterly follow-up with the surgeon and 35% every six months, with only 10.7% holding monthly consultations. With the nutritionist the rate of quarterly visits was similar (35.9%), followed by month (32%) and six months (13.6%). Now with psychologist, 29.1% of participants did not perform any visit, 15.5% attended biweekly and 13.6% monthly. Frequency similar to the psychologist, 31% reported they did not return for follow-up with endocrinologist, but 13.6% did quarterly monitoring and 15.5% every six months. Regarding the post-surgery, the missing data rate on compliance with the endocrinologist (26.2%) and psychologist (24.3%) was high. Other professionals consulted during this period were: psychiatrist, physiotherapist, hematologist, cardiologist, orthopedist, gynecologist and dermatologist.

**FIGURE 3:**
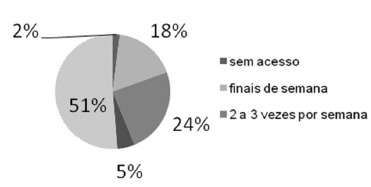
- Internet usage frequency to search for information about health and bariatric surgery (n=103)

Responses to EQ showed that 32% (n=33) of participants reported difficulty maintaining multi-professional assistance. Among the reported reasons included: high cost to consult with professionals and exams, when not covered by health agreement; lack of time; unavailability agenda of the surgeon; and do not consider important. When asked about the use of the internet (Figure 3), 51.5% (n=53) of respondents accessed the internet for information on health and bariatric surgery every day, compared to only 2% who did not used it with this intention.

As shown in Table 2, Facebook and search tools were appointed as the search sites most used for information, followed by sites dedicated to disseminating information on bariatric surgery. Other places mentioned in the EQ were bases of scientific papers, communities and groups created by patients after bariatric surgery, reports in newspapers and magazines online. Asked whether they had other sources of information on bariatric surgery than the internet, 74% (n=76) responded "No" and 26% (n=27) "Yes."

**TABLE 2:** - Favorite places to search for information on the internet (n=103)

	**n**	**%**
Facebook	83	80,6
Search engines (Google, Yahoo)	72	69,9
Service sites in which it is assisted	39	37,9
Sites which centralize information on bariatric surgery	37	35,9
Sites of other services that provide assistance to bariatric patients	35	34,0
Blogs/fotologs	33	32,0
E-mails received from professionals who accompanied the patient in the preoperative	15	14,6
Sites with less technical terms	6	5,8

The most sought after information on the internet were about bariatric surgery weight loss, healthy eating and weight regained (Table 2). On the other hand, thematic least accessible were associated with possible difficulties during and after surgery, including the latest research on bariatric surgery and possible complications after its completion.

**TABLE 3:** - Type and frequency of information sought on the internet (n=103)

**Thematic sought on the internet**	**n**	**%**
Weight loss after bariatric surgery	79	76,7
Healthy eating	79	76,7
Regained weight	71	68,9
Postoperative care	66	64,1
Physical activity	64	62,1
Plastic surgery	62	60,2
Spaces to interact with other bariatric patients	61	59,2
Latest research on bariatric surgery	36	35,0
Complications after bariatric surgery	29	28,2

In addition to the search for information 84% of participants (n=86) stated that they have participated in any social network related to bariatric surgery. In respect of such participation, they cited the following benefits: experience exchange possibility; connect and interact with other bariatric patients; give and receive support; access to information; able to answer questions.

Analyzing the relationship between participation in social networks and follow-up with the multidisciplinary team, identified negative correlation (r= -0.23, p=0.024) between such participation and follow-up with a nutritionist, showing that, when participating in social networks, patients tend to decrease or interrupt the action with the nutritionist.

**FIGURE 4:**
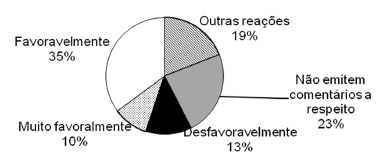
- Perception of patients on the reaction of professionals when informed about the search for information on the internet

Assessing the influence of internet use in communication between patients and health professionals, it was found that the majority (72%) commented with professionals on the search for information about bariatric surgery on the internet. In light of these reports (Figure 4) 45% (n=46) of the professionals responded favorably to very favorable; 23% (n=24) did not comment; 13% (n=13) unfavorably.

Analyzing the relationship between communication about the patient make use of information from the internet and the reaction of professionals regarding this report, it was identified a significant negative correlation (r= -0.71, p=0.001), showing that the professional trend is to react negatively when they are informed the patient went to internet to seek for information.

To increase knowledge on how to reduce the loss of follow-up, participants were asked to indicate strategies that could be used by professionals to stimulate follow-up after surgery. In this sense, they answered: a) monthly meetings for guidance - 58% (n=56); b) asking monthly group presence support - 52% (n=54); c) invitation to walking groups - 27% (n=28) weekly and 27% (n=28) monthly; d) provision of occupational therapy - 17% (n=18) weekly and 33% (n=34) monthly; e) offer virtual support groups - 41% (n=42) weekly and 27% (n=26) monthly.

## DISCUSSION

In general, the adhesion to the multidisciplinary follow-up after the operation breaks down. Despite not having been found empirical studies that presented data on this subject in Brazil, there are reports of several authors on factors that may negatively influence following the multidisciplinary follow-up in this period.

In the present study, was analyzed another factor quite evident in the modern world: the use of internet. The participants, mostly reported that they used the internet enough to access information about their health and bariatric surgery. In this regard, it was inferred that the high frequency of use can negatively impact following since daily access puts the patient in contact with large amounts of information and it can begin to consider unnecessary care by a professional. Depending on the type of access he/she may even have met their psychosocial needs as to feel accepted and understood, maintain social interaction and receive reinforcing stimuli for weight loss.

It is noteworthy that the literature has pointed to the increasing demand for information online instead of medical appointments, as well as the replacement of medical care or the information provided by health professionals for the information the internet^3^^,^ 17 ^,^ 18. Is also noteworthy, that the majority of participants also participated in social network related to bariatric surgery, which reflects the growing role that social networking has taken to many patients, not only as a search resource for information, but also for interactions with similar people, looking for social support, fostering hope and quality of life^7^^,^ 12. Still a high number of participants (74%) declared that had other sources of information about bariatric surgery beyond the internet. Such patients seemed to think internet has all the knowledge, because it allows almost unlimited access to more diverse sources of information, scientific or not, and allows to publicize what they wish to their peers and their social support network.

The EQ revealed that the majority of participants (72%) commented to professionals seeking information on bariatric surgery on the internet. However, 13% still reacted unfavorably and 23% did not make comments about it. In this sense, it is noteworthy the result of the identified correlation, which indicates that professionals tend to react unfavorably when the patient indicates that sought information on the internet. This finding highlights the presence of professionals unprepared to deal with the increasing use of this tool. The literature has shown that patients avoid comment on the use of the internet with professionals, mainly for fear that this would interfere with the relationship between them and the professional, and he should interpret such behavior as a lack of confidence in the care^3^.

The literature also points out that many professionals end up reacting negatively to concerns about the reliability of information that patients get on the network, because they feel tested by patients or bothered for not knowing the information presented and even the risk that the consultation extend more than expected^19^. There are also those who can fidget with the possibility to occur replacement of care or medical guidelines for internet information. Last but not least, many professionals fear the occurrence of possible somatization symptoms by misunderstanding of the information or access to false information^3^^,^ 5 ^,^ 11 ^,^ 12 ^,^ 17 ^,^ 18 ^,^ 19.

Given the above, it is clear that the potential use of the internet in health has to modify the relationship between professional and patient. In a study^5^ of 116 medical teachers, it was found that 56.9% of respondents believed the internet helped in the doctor-patient relationship, 27.6% thought they did not interfere and 15.5% believed that the internet hindered. For respondents favorable to its proper use, the information accessed can facilitate knowledge about the disease, improve their adherence to treatment and help share the responsibility for decision-making, assisting in the development of better communication and professional-patient relationship. Already unfavorable was highlighted the inappropriate use of information obtained on the internet, stating that the doctor-patient relationship can be undermined by access to incorrect or difficult to interpret information. Also signaled the possibility of patients have somatic symptoms and maladaptive psychological reactions and even go to perform self-medication. With regard to the feelings of physicians when patients mentioned information acquired on the internet, the authors found that 14% indicated mixed feelings and 11% confirmed uncomfortable feeling.

However, it is emphasized that informed patient no means prepared patient, since often the information alone is not enough to promote the necessary behavioral changes. To patient be properly prepared, healthcare professionals need to learn to deal with this new context, which includes the internet among the main sources of information. Professional must be prepared to deal with behavioral changes that patients are experiencing and help them make the best use of information available on the network^3^. It is necessary to learn to explore the use of the internet with the patients. To this end, it is suggested that during the consultations: a) include questions to assess the type of use that the patient makes the network by checking the main sources of information on health and bariatric surgery access, the frequency of searching for information, reliability of information accessed, participation in social networks about bariatric surgery and the benefits perceived by patients with the use of information obtained on the internet; b) recommend trusted sites; c) provide additional information and explanations to the information reported by them; d) validate the feelings and behaviors that led them to seek information on the internet.

Another aspect relates to the better use of the network by patients and health professionals. The search tools on the internet most used by the participants was similar to that identified in other studies^1^^,^ 3 ^,^ 17 ^,^ 19, the sites of the institutions in which the patient is assisted as well as those that centralize the information on bariatric surgery, such as the Brazilian Society for Bariatric and Metabolic Surgery. They are used less than Facebook and research tools. Among the possible explanations for this fact may be mentioned: a) lack of recommendation of the own professionals about the site information; b) outdated content; c) low attractiveness of institutional sites compared to other areas, such as social networking patient groups. Draws attention the limited investment in upgrading the institutional sites, since there is finance expense for the creation and maintenance of these vehicles. To keep such sites more attractive and updated, it is necessary to foster the interest of traders and make them aware of the importance of this vehicle to improve the relationship and communication with patients. It is suggested that such sites be actively updated and ask to the patients information how interesting they are. According to the results of this study the information of greatest interest are: a) aspects related weight loss after bariatric surgery; b) guidelines for healthy eating; c) clarification and guidance to prevent weight regained.

In view of the data presented, it is clear that many professionals are still not prepared to discuss with patients the information they access the internet. However, there is no escaping the obvious technological advance in terms of easier access to information. It is therefore important that professionals are prepared to discuss the information that patients get on the internet, to clarify and help them understand what they are reading and watching. The content can be used as an ally in building better bond with patients, helping them in decision, making and fostering active participation in treatment. If the professionals explore the use of the internet to their patients in order to facilitate communication with them, it can stimulate greater engagement in the changes required by the treatment. Recommend trusted sites, discuss information, participate more actively in open or institutional sites and social networks, providing reliable information, and contribute to the communication, stimulate the follow-up in the postoperative period.

This study achieved its goals, but the number of patients is too small for generalized the results to the entire population of bariatric patients. Multicenter studies are needed to evaluate the use of the internet and its relationship with the patient follow-up, including different Brazilian states and patients from different socioeconomic groups. EQ was the chosen way for dissemination, data collection and recruitment of participants. Effectively counted on the opinions of internet users, not getting information from those who have less access to the network, but still make some use of such informative way. The discussion of the data was limited to national studies on the subject, because there are significant differences in internet usage profile among Brazilian patients and those of other countries, especially the more developed. Studies comparing this reality could help to better understand what strategies can be used to turn the internet an ally in long follow-up after bariatric surgery.

## CONCLUSION

The information in the internet influence the multidisciplinary follow-up, a fact that requires staff to consider using the network as a variable that interferes and must be managed. It is suggested the active participation of the professionals on their websites and social networking, and the diversification of interventions and services to stimulate long follow-up after the operation.
